# Impact of neoadjuvant chemotherapy on the association between key genetic alterations and tumor microenvironment in upper tract urothelial carcinoma

**DOI:** 10.1007/s00262-026-04500-1

**Published:** 2026-07-27

**Authors:** Daiki Ikarashi, Kenta Takahashi, Shigehisa Kitano, Daichi Tamura, Masakazu Abe, Ayato Ito, Ei Shiomi, Shigekatsu Maekawa, Renpei Kato, Mitsugu Kanehira, Naoki Yanagawa, Wataru Obara

**Affiliations:** 1 Department of Urology, Iwate Medical University School of Medicine, Iwate, 028-3695 Japan; 2 Division of Cancer Immunotherapy Development, Department of Advanced Medical Development, The Cancer Institute Hospital of Japanese Foundation for Cancer Research, Tokyo, 135-8550 Japan; 3 Division of Clinical Chemotherapy, Cancer Chemotherapy Center, Japanese Foundation for Cancer Research, Tokyo, 135-8550 Japan; 4 Department of Pathology, Iwate Medical University School of Medicine, Iwate, 028-3695 Japan

**Keywords:** Upper tract urothelial carcinoma, *TP53*, *FGFR3*, *RAS*, Tumor microenvironment, NAC

## Abstract

**Background and Objective:**

Adjuvant nivolumab has demonstrated clinical benefit in urothelial carcinoma; however, emerging evidence suggests that its efficacy may differ between bladder cancer and upper tract urothelial carcinoma (UTUC), potentially reflecting differences in genomic profiles and the tumor microenvironment (TME). This study aimed to evaluate the association between key oncogenic mutations and the TME characteristics in UTUC, with a particular focus on the impact of neoadjuvant chemotherapy (NAC).

**Methods:**

We analyzed tumor specimens from 32 patients who underwent nephroureterectomy for UTUC. Patients were categorized into NAC and non-NAC groups. Oncogenic mutations (*TP53*, *FGFR3*, and *RAS*) were assessed in all patients, and immune cell densities (CD3^+^, CD4^+^, CD8^+^, Foxp3^+^, and CD204^+^) in intratumoral and peritumoral regions were quantified using multiplex fluorescent immunohistochemistry.

**Results:**

*TP53*, *FGFR3*, and *RAS* mutations were identified in 29%, 31%, and 34% of patients, respectively. NAC was administered to 22% of patients. The NAC group demonstrated significantly higher densities of intratumoral CD4^+^ T cells (*p* = 0.0201) and a trend toward increased CD8^+^ T cells (*p* = 0.0614). Among patients with *TP53* mutations, NAC was associated with significantly higher intratumoral CD4^+^ T cell density (*p* = 0.0369) compared without NAC. In contrast, *FGFR3*-mutant tumors exhibited consistently low immune cell infiltration, irrespective of NAC status.

**Conclusions:**

*TP53*-mutant tumors from patients who received NAC were associated with higher intratumoral CD4⁺ T cell density, whereas *FGFR3*-mutant tumors exhibited persistently low immune-cell infiltration irrespective of NAC exposure. These findings highlighted distinct mutation-associated TME features in UTUC.

**Supplementary Information:**

The online version contains supplementary material available at https://doi.org/10.1007/s00262-026-04500-1.

## Introduction

Urothelial carcinoma (UC) has been recently classified at the molecular level, including a comprehensive analysis conducted on oncogene mutations and immune activity in the tumor microenvironment (TME) [1]. The association between oncogene mutations and the TME in upper tract UC (UTUC), particularly in the changes in neoadjuvant chemotherapy (NAC), remains unclear. The CheckMate 274 trial has recently demonstrated the clinical efficacy of adjuvant therapy with nivolumab as an immune checkpoint inhibitor (ICI) [2]. However, the effect of adjuvant nivolumab was limited in UTUC compared with bladder cancer. Interestingly, subgroup analyses suggested that patients with UTUC who had received NAC derived greater benefit from adjuvant nivolumab than those who had not received NAC [2].

UTUC exhibits molecular characteristics distinct from bladder cancer, including a higher prevalence of FGFR3 alterations and a predominantly luminal phenotype, both of which have been associated with reduced immune infiltration [1, 3]. Distinct genomic profiles of UTUC and the changes in the TME following NAC may underlie this disparity. We previously reported the association between key oncogenic alterations in UTUC and several immune component cells in the TME; however, cases of NAC were excluded [4]. Moreover, our previous study in muscle-invasive bladder cancer has demonstrated that platinum-based NAC can increase tumor-infiltrating lymphocytes and modulate antitumor immune responses in the TME [5]. We hypothesized that NAC-induced changes in the TME differ according to the genomic background of UTUC and may contribute to the heterogeneous efficacy of adjuvant immunotherapy observed in this disease. Therefore, this study aimed to investigate the clinical significance of the association between the TME characteristics and the key oncogene mutations in patients with UTUC with or without NAC.

## Materials and methods

### Patient

We retrospectively analyzed 45 patients who underwent nephroureterectomy for UTUC and oncogene mutation analysis by surgical specimen at Iwate Medical University from January 2012 to July 2022. A flowchart of the patient selection process is shown in Supplemental Fig. 1. Four patients with no histological findings of malignancy and nine patients with insufficient analysis specimens were excluded. Finally, this study included 32 eligible patients, who were subsequently categorized into two groups based on platinum-based NAC: the NAC group (*n* = 7) and the non-NAC group (*n* = 25). The indications for NAC were locally advanced high‐risk UTUC, including cT3–4 and/or cN + diseases. NAC consisted of gemcitabine plus cisplatin (GC) or carboplatin (GCa), administered for 2–3 cycles.

## Genomic characterization

Whole-exome sequencing of tumor tissue DNA was performed using target capture with SureSelect XT Human All Exon v6 (Agilent Technologies, Santa Clara, CA, USA), as previously described [4, 6]. The genetic characterization of UTUC was performed by analyzing mutations frequently reported in UC, specifically tumor protein 53 (*TP53)*, fibroblast growth factor receptor 3 (*FGFR3)*, and *RAS* (*HRAS*, *KRAS*, and *NRAS*) [1].

### Tissue sample selection and multiplex fluorescence immunohistochemistry

After reviewing the hematoxylin and eosin (H&E)-stained slides from the surgical resection specimens by a pathologist (N.Y.), a representative tissue block was selected for each case. Tumor cores measuring 6.0 mm in diameter were then obtained from the center of the tumor nest within the selected block using a manual tissue arrayer (KIN-2; Azumaya, Japan), and tissue microarrays (TMAs) were constructed. The TMA blocks were sectioned at a thickness of 4 μm for multiplex fluorescent immunohistochemistry. The summary of mFIHC is described below following previous studies [4, 6]. We used the following markers for staining: CD3, CD4, CD8, forkhead box protein 3 (FoxP3), CD204, and CK. The entire slide of each core was scanned at × 100 using an automated imaging system (Vectra Polaris; AKOYA Biosciences, Marlborough, MA, USA). Various immune cell densities in the TMA were calculated as follows: counts of immune cells/intratumoral or peritumoral area (mm^2^). The intra- and peritumoral areas were selected from the area where the tumor was viable and bordering the tumor, respectively, in the TMA (Supplemental Fig. 2).

## Statistical analysis

The Wilcoxon rank-sum test (Mann–Whitney U test) was employed for analyzing differences in densities of each immune cell based on NAC status and genetic mutations. To evaluate the combined effects of genomic mutations and NAC, patients were stratified according to TP53 or FGFR3 mutation status and NAC exposure. Comparisons among the four groups were performed using the Kruskal–Wallis test followed by Steel–Dwass post hoc multiple-comparison testing. In addition, exploratory interaction analyses were performed to assess whether the association between NAC and immune cell density differed according to mutation status. The Kaplan–Meier method was used for calculating progression-free survival (PFS) and overall survival (OS). All statistical analyses were performed using JMP 14.0 software (SAS Institute Inc., Cary, NC, USA). Given the exploratory nature of this study and the limited sample size, adjustments for multiple comparisons were not performed. Therefore, all statistical findings should be interpreted as hypothesis-generating. Differences with *p* < 0.05 were considered statistically significant for all statistical comparisons.

## Result

### Patients’ characteristics

The patients’ characteristics are presented in Table 1. All patients were treated with curative intent and received two or three cycles of NAC, followed nephroureterectomy. NAC was administered to 22% of patients. There were no significant differences between NAC and non-NAC groups.

**Table 1:** Patient characteristics

Variables		Total (n = 32)	NAC status		*p *value
			NAC group (n = 7)	non-NAC group (n = 25)	
Age (Median, range)		72 (55–82)	71(59–76)	72(55–82)	*p* = 0.3107
Sex (No.)	M	25 (78%)	5	20	*p *= 0.6355
	F	7 (22%)	2	5	
Lt/Rt	Lt	18 (56%)	4	14	*p* = 0.9570
	Rt	14 (44%)	3	11	
Primary site	pelvis	17 (53%)	2	15	*p* = 0.1371
	ureter	15 (47%)	5	10	
pT stage	pT2≧	13 (41%)	4	9	*p* = 0.7419
	pT3≦	19 (59%)	3	16	
Recurrence	+	17 (53%)	2	15	*p* = 0.1371
	-	15 (47%)	5	10	
Gene mutation*	FGFR3	10 (31%)	2	8	*p* = 0.8619
	TP53	9 (28%)	3	6	*p* = 0.3267
	RAS	11 (34%)	0	11	–

NAC; neoadjuvant chemotherapy

*Percentages were calculated based on the full cohort (n = 32). Three patients harbored mutations in more than one of these genes (including one patient with mutations in all three genes), and six patients had mutations in genes other than FGFR3, TP53, or RAS

## Genomic and immunological findings

Genetic mutation analysis revealed *TP53*, *FGFR3*, and *RAS* mutations in 9 (29%), 10 (31%), and 11 patients (34%), respectively. Three patients harbored mutations in more than one of these genes, including one patient with mutations in all three (*FGFR3*, *TP53*, *RAS*) genes. In addition, six patients had no mutations in *FGFR3*, *TP53*, or *RAS* but harbored mutations in other genes. In the NAC group (*n* = 7), 3 (43%), 2 (29%), and no patients demonstrated *TP53*, *FGFR3*, and *RAS* mutations, respectively. Conversely, in the non-NAC group (*n* = 25), *TP53*, *FGFR3*, and *RAS* mutations were identified in 6 (24%), 8 (32%), and 11 patients (44%), respectively. PFS and OS were analyzed according to each genetic mutation status to evaluate the prognostic impact of oncogenic alterations. Patients with *TP53*-mutant tumors exhibited a significantly shorter PFS than those with *TP53* wild-type tumors (*p* = 0.0184). By contrast, no significant difference in PFS was observed between patients with and without *FGFR3*/*RAS* mutations. Moreover, no significant difference in OS according to each genetic mutation status was observed (Supplemental Fig. 3).

**Fig. 1:**
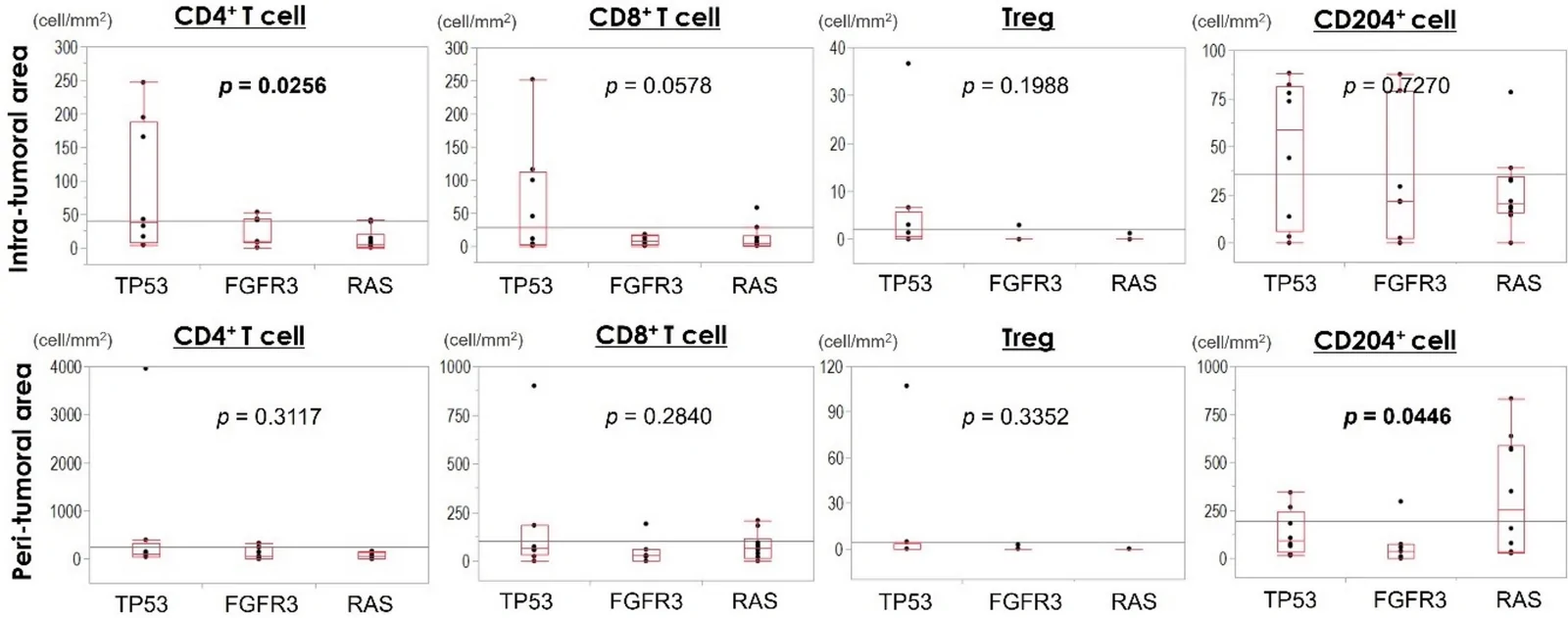
Assessment of the density of each immune cell type in the tumor immune microenvironment among *TP53*-, *FGFR3*-, and *RAS*-mutated tumors Differences in the density of each immune cell type in the intratumoral and peritumoral areas among TP53-, FGFR3-, and RAS-mutated tumors were analyzed using analysis of variance. Intratumoral CD4 ^+^ T-cell density and peritumoral CD204^ +^ cell density differed significantly among these groups (intratumoral CD4^ +^ T cell: *p* = 0.0256, peritumoral CD204 ^+^ cell: *p *= 0.0446)

Subsequently, using Kruskal–Wallis test, we assessed the differences in each immune cell infiltration among the *TP53*-, *FGFR3*-, and *RAS*-mutated groups. Consequently, differences among the mutational groups were observed in intratumoral CD4⁺ T cell infiltration (*p* = 0.0256) and peritumoral CD204⁺ cell infiltration (*p* = 0.0446). Overall, *TP53-*mutated tumors tended to exhibit higher levels of immune cell infiltration, whereas *FGFR3*-mutated tumors generally showed lower levels of immune cell infiltration, although only selected immune cell populations reached statistical significance (Fig. 1).

## TME stratified by NAC and gene mutation status

**Fig. 2:**
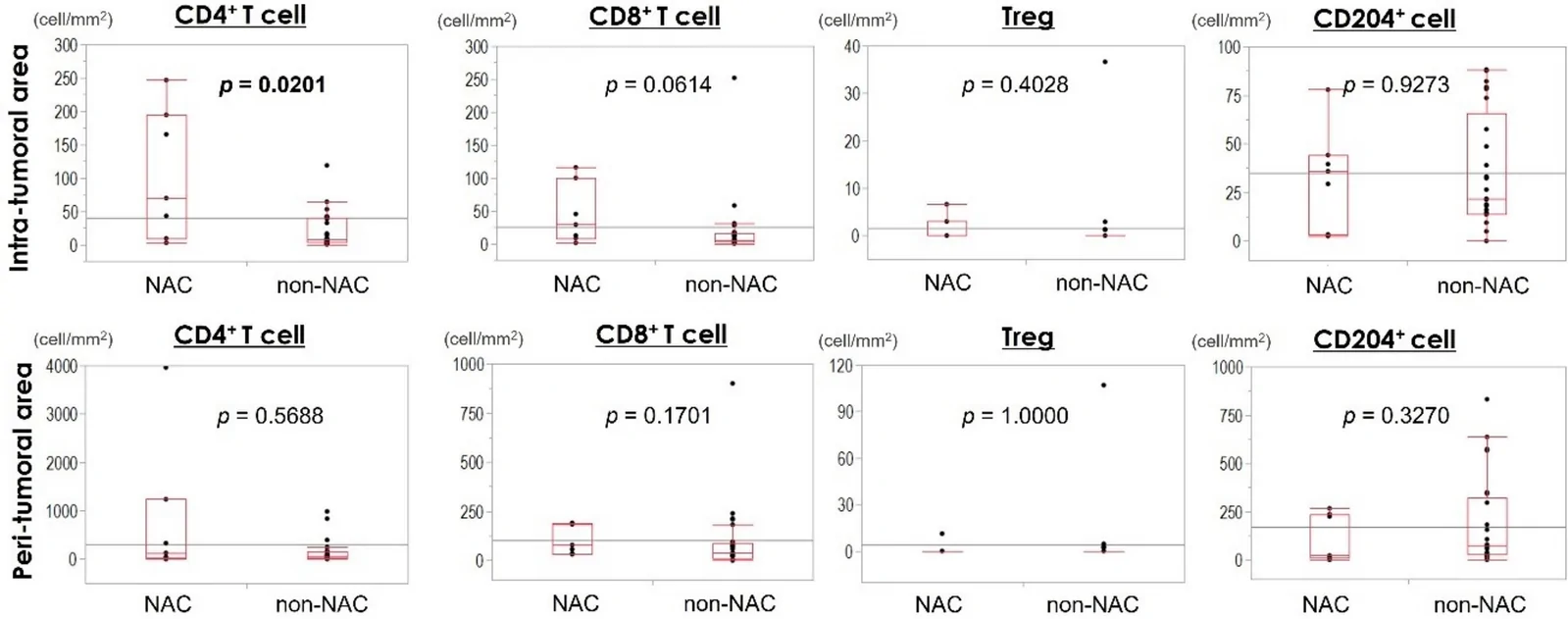
Association between the density of each immune cell type in the tumor immune microenvironment and the presence or absence of neoadjuvant chemotherapy (NAC) in this study Relationship of the density of each immune cell type in the intratumoral and peritumoral areas between the NAC and non-NAC groups. The density of intratumoral CD4 ^+^ T cells was significantly higher in NAC group compared with non-NAC group (*p* = 0.0201)

First, the association with NAC status and immune cell infiltration in the TME was evaluated in patients with UTUC regardless of specific gene mutations. The NAC group showed significantly higher densities of intratumoral CD4^+^ T cells (*p* = 0.0201) and a trend toward higher densities of intratumoral CD8^+^ T cells (*p* = 0.0614) than the non-NAC group. Other immune cells showed no significant difference between NAC and non-NAC groups (Fig. 2).

**Fig. 3:**
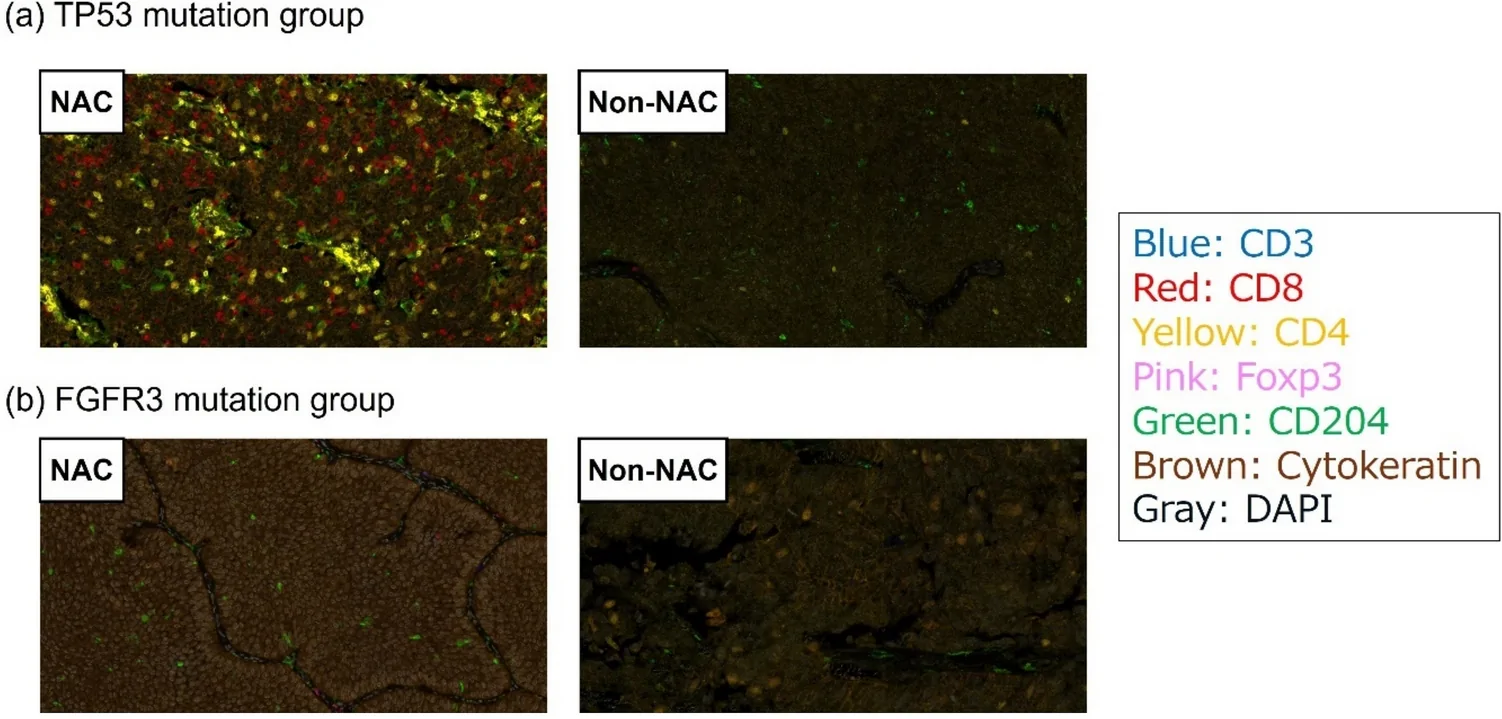
Comparison of representative mFIHC images by NAC status in *TP53*- and *FGFR3*- mutated tumors Representative image of multiplex fluorescence immunohistochemistry of surgical specimens with or without NAC in (a) TP53-mutated and (b) FGFR3-mutated tumors for CD3, CD4, CD8, CD204, FoxP3, cytokeratin, and 4′,6-diamidino-2-phenylindole. Original magnification, × 200. Multiplex immunofluorescence signals are visualized using the following color scheme: CD3 (blue), CD4 (yellow), CD8 (red), Foxp3 (pink), CD204 (green), cytokeratin (CK; orange), and DAPI (gray). Representative images illustrating typical staining patterns observed in each group

**Fig. 4:**
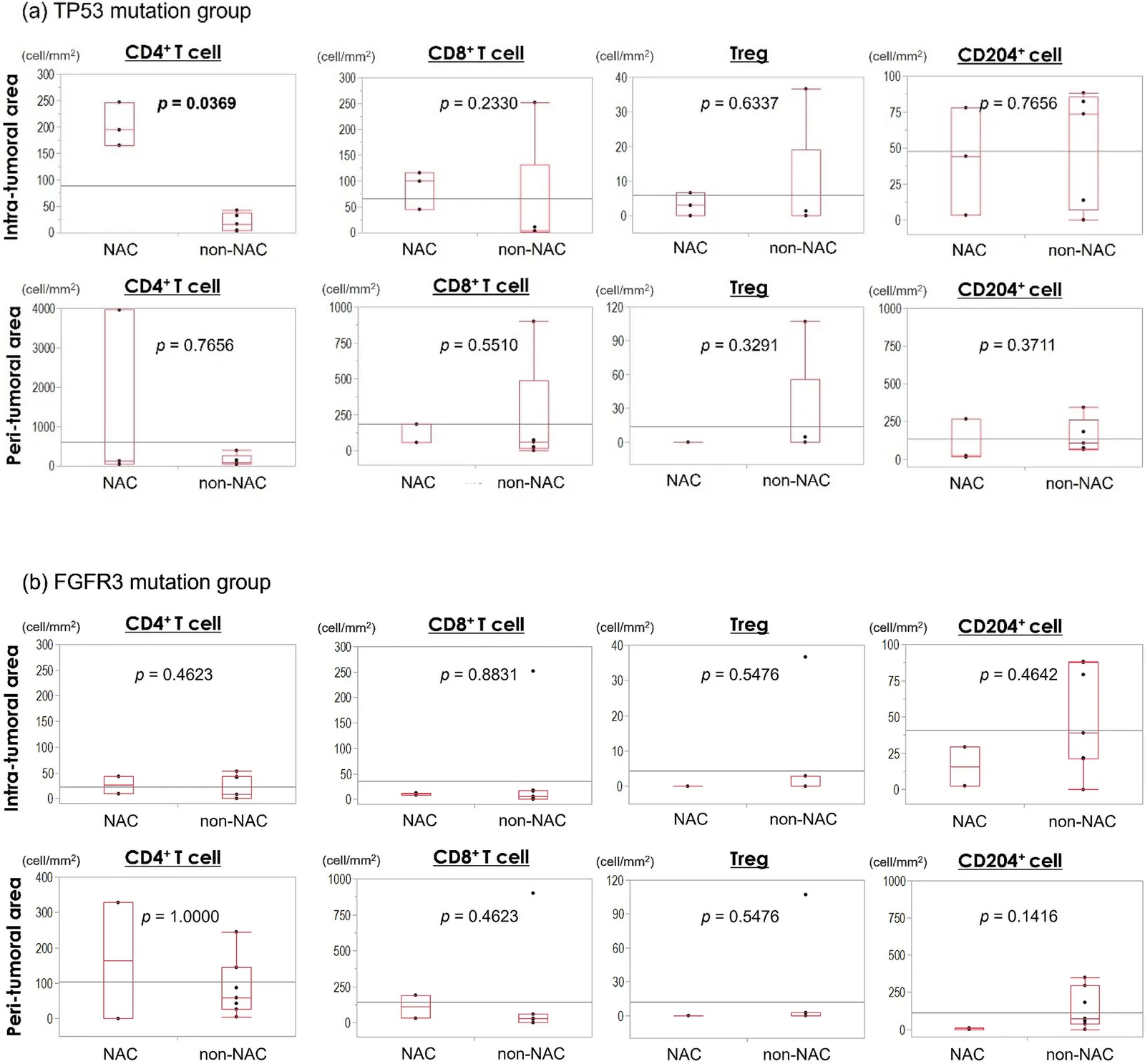
Immune cell infiltration according to NAC status in TP53- and FGFR3-mutated tumors Association between the density of each immune cell type in the intratumoral and peritumoral areas between the (a) TP53-mutated and (b) FGFR3-mutated groups. Intratumoral CD4^ +^ T cell density is significantly higher with NAC than the non-NAC specimen in the TP53-mutated group (*p* = 0.0369). No significant differences are noted between NAC and non-NAC specimens in the FGFR3-mutated group

**Fig. 5:**
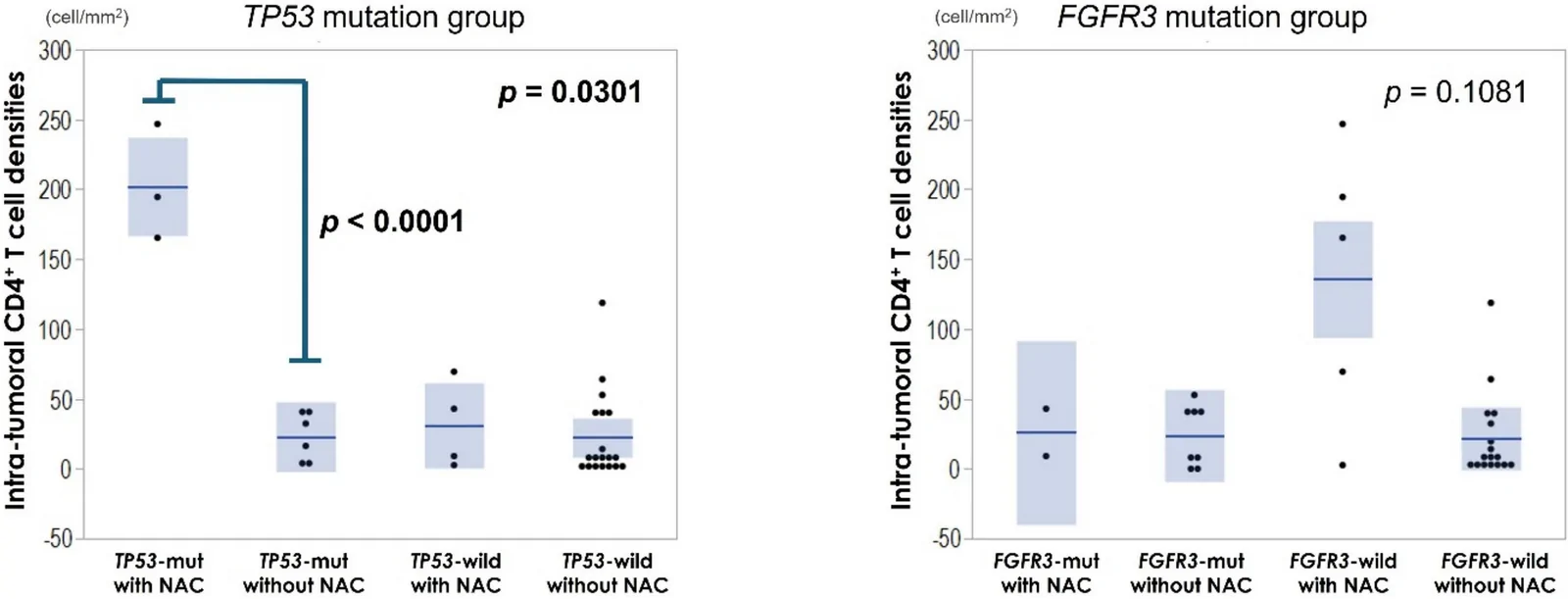
Four-group analysis according to TP53/FGFR3 mutation status and NAC exposure for intratumoral CD4^+^ T cell density Individual data points are shown. Patients were stratified according to TP53 or FGFR3 mutation status and NAC exposure. Comparisons among the four groups were performed using the Kruskal–Wallis test followed by post hoc multiple-comparison testing. A significant overall difference was observed among the TP53/NAC subgroups (*p* = 0.0301), whereas no significant difference was observed among the FGFR3/NAC subgroups (*p* = 0.1081)

Next, we investigated immune cell infiltration in the TME according to NAC status in patients with *TP53* or *FGFR3* mutations who received NAC via mFIHC (Fig. 3). Patients with *TP53* mutations showed significantly higher densities of intratumoral CD4^+^ T cells (*p* = 0.0369) in the NAC group than non-NAC group. Conversely, patients with *FGFR3* mutations demonstrated lower densities of each immune cell as a cold tumor with or without NAC (Fig. 4). As an exploratory analysis, patients were stratified according to *TP53* or *FGFR3* mutation status and NAC exposure to investigate the combined effects of genomic alterations and NAC on the tumor immune microenvironment. Among *TP53*-mutant tumors, a significant overall difference in intratumoral CD4^+^ T cell density was observed among the four groups (Kruskal–Wallis test, *p* = 0.0301). Post hoc analysis also revealed that *TP53*-mutant tumors treated with NAC exhibited significantly higher intratumoral CD4^+^ T cell density than *TP53*-mutant tumors without NAC. On the other hand, *FGFR3*-mutant tumors generally exhibited lower immune cell infiltration irrespective of NAC status, while relatively higher immune cell densities were observed in *FGFR3*-wild-type tumors (Fig. 5). Furthermore, exploratory interaction analysis suggested a potential interaction between *TP53* mutation status and NAC exposure with respect to intratumoral CD4^+^ T cell density, indicating that the effect of NAC differed according to *TP53* mutation status. In contrast, no significant interaction was observed between *FGFR3* mutation status and NAC exposure, although *FGFR3*-mutant tumors consistently exhibited low levels of immune cell infiltration irrespective of NAC status (Supplementary Fig. 4).

In addition, to investigate a potential mechanism underlying the increased immune cell infiltration observed after NAC, single immunohistochemical staining for HMGB1, a marker associated with immunogenic cell death, was performed according to NAC status. HMGB1 expression was observed in NAC-treated tumors, whereas little or no expression was detected in tumors from patients who did not receive NAC (Supplementary Fig. 5).

## Discussion

This exploratory study suggests that UTUC exhibits distinct TME characteristics depending on oncogenic mutation status. *FGFR3*-mutated tumors were associated with an immune-cold phenotype, whereas *TP53*-mutated tumors tended to exhibit features of a more immune-enriched microenvironment, particularly following NAC. These findings align with prior transcriptomic studies indicating reduced immune infiltration and interferon signaling in *FGFR3*-mutated UC [7, 8]. To our knowledge, this study is the first to explore a key oncogenic mutation stratified and NAC-based comparative analysis of TMEs in UTUC, using mFIHC for spatially resolved protein expression profile.

The persistence of immune-cold features following NAC in *FGFR3*-mutated tumors suggests intrinsic resistance to immune activation. Chemotherapy induces immunogenic cell death (ICD) and enhances T cell recruitment [9]; however, such effects appeared limited in *FGFR3*-mutated tumors in our cohort. This molecularly defined immune heterogeneity potentially contributes to the modest ICI benefit observed in UTUC compared with bladder cancer in the CheckMate 274 trial [2].

Clinically, incorporating molecular and immune profiling could refine patient selection for adjuvant immunotherapy. *FGFR3* mutation status may inform personalized treatment strategies identifying patients who are more likely to benefit from alternative or combination strategies, including FGFR inhibitors plus ICIs [10]. Furthermore, understanding the genomic-immune interplay may guide the timing of systemic therapies and future biomarker-driven trials in UTUC.

Contrary to *FGFR3*-mutated tumors, *TP53*-mutated tumors are frequently characterized by a T cell–inflamed phenotype with limited immune cell infiltration. The immune-enriched microenvironment observed in *TP53*-mutated tumors may therefore reflect fundamental differences in tumor biology, including genomic instability and inflammatory signaling. Moreover, in *TP53*-mutated UTUC, the impact of NAC on the spatial distribution of immune cell infiltration within the TME remains largely unclear. Our study revealed that NAC patients with *TP53* mutations exhibited significantly higher densities of intratumoral CD4^+^ T cells than the non-NAC group. One possible explanation for this observation is the involvement of immunogenic cell death (ICD)-related pathways, which have been reported to be associated with the release of damage-associated molecular patterns, including HMGB1, following cytotoxic chemotherapy [11]. Extracellular HMGB1 has been shown to stimulate dendritic cells through toll-like receptor 4 signaling, thereby enhancing antigen processing and presentation [12].

In tumors harboring *TP53* mutations, defective DNA damage response pathways and impaired cell-cycle checkpoint control may amplify chemotherapy-induced ICD, causing heightened HMGB1 release and augmented immune activation [13]. Consistent with this concept, higher intratumoral CD4⁺ T cell infiltration observed following NAC in *TP53*-mutated tumors suggest HMGB1-mediated improved local antigen presentation and immune orchestration. Along with these results, our findings suggest that by promoting CD4⁺ T cell-dominant immune infiltration through ICD-associated pathways, NAC exerts immunomodulatory effects beyond direct cytotoxicity, particularly in *TP53*-mutated tumors. Collectively, these findings suggest an association between NAC exposure and higher intratumoral CD4⁺ T-cell density in *TP53*-mutated UTUC. Nevertheless, the present data do not establish causality, and further studies incorporating paired specimens and functional analyses are required to clarify the underlying biological mechanisms.

This study has several limitations, with a small sample size, and retrospective analysis. In particular, the *TP53* or *FGFR3* mutation and NAC status subgroup included only a small number of cases, which may have reduced statistical power and increased the risk of statistical instability. In addition, multiple subgroup comparisons were performed across several immune cell populations, and formal correction for multiple testing was not applied because of the exploratory nature of this study. Therefore, the possibility of type I error cannot be excluded. Nevertheless, these findings should be considered hypothesis-generating and require validation in larger independent cohorts. Furthermore, the specimens analyzed were not paired samples (i.e., they were not matched pre- and post-NAC UTUC samples). Nevertheless, tumor biopsy is not performed in routine clinical practice for UTUC as it necessitates an ureteroscope examination, which is relatively invasive. Moreover, comparisons between the NAC and non-NAC groups are potentially subject to selection bias. NAC administration was not randomized and may have been influenced by clinical factors such as patient fitness, renal function, disease burden, and physician preference. Although no significant differences in baseline characteristics were observed, the limited sample size may have reduced the ability to detect clinically meaningful imbalances between groups. Therefore, the observed differences in immune cell infiltration cannot be attributed solely to NAC exposure and should be interpreted with caution. In addition, gene mutation categories were not mutually exclusive, as some patients harbored mutations in more than one of the genes analyzed, including one patient with mutations in all three genes. Although *TP53* and *FGFR3* alterations are often associated with distinct molecular subtypes of urothelial carcinoma, co-occurring genomic alterations have been reported in both bladder cancer and UTUC [14, 15]. Therefore, the immune microenvironmental characteristics observed in the present study cannot be attributed exclusively to individual genomic alterations, and the potential influence of co-occurring mutations should be considered when interpreting the results. Despite these limitations, we believe that our findings that the TME differ markedly according to key genetic alternations depending on NAC status are supported by previously established methods from our group [3, 5, 16] and offer hypothesis-generating evidence supporting the biological distinctiveness of UTUC compared with bladder cancer.

In conclusion, our findings suggest that UTUC may exhibit mutation-specific immune landscapes: *FGFR3*-mutated tumors tending to be immune-cold, whereas *TP53*-mutated tumors tended to exhibit features of a more immune-enriched microenvironment. NAC might modulate the TME; however, the effects appear mutation dependent. These observations provide a rationale for further investigation of mutation-informed therapeutic strategies and integrated molecular–immune profiling in UTUC. Further prospective studies are warranted to validate these observations and to optimize stratified therapeutic approaches.

## Supplementary Information

Below is the link to the electronic supplementary material.

Supplementary file1 (DOCX 19 KB)

Supplementary file2 (PPTX 36774 KB)

## Data Availability

No datasets were generated or analyzed during the current study.
