# Supplementary material for: Impact of neoadjuvant chemotherapy on the association between key genetic alterations and tumor microenvironment in upper tract urothelial carcinoma
Source: Cancer Immunol Immunother. 2026 Jul 27;75(11):255. doi: 10.1007/s00262-026-04500-1 (PMC13710514; doi:10.1007/s00262-026-04500-1)
Supplement: Supplementary file 1 [file 262_2026_4500_MOESM1_ESM.docx]

**Cancer Immunology, Immunotherapy (submitted in 2026) - Daiki Ikarashi et al.**

**Supplemental Materials**

**Supplemental Fig. 1** Flow diagram of patients included in the study

**Supplemental Fig. 2** Representative image of tissue segmentation

Tissue segmentation was performed using inForm software. Tumor regions (red) and stromal/peritumoral regions (green) were identified using a machine-learning–based tissue classification algorithm. Immune-cell densities were quantified separately within intratumoral and peritumoral compartments.

**Supplemental Fig. 3** Prognosis following RNU

Kaplan–Meier curves of (a) PFS and (b) OS stratified by *TP53*-, *FGFR3*-, and *RAS*-mutated groups following RNU for UTUC. PFS, progression-free survival; OS, overall survival; RNU, radical nephroureterectomy; UTUC, upper tract urothelial carcinoma

**Supplemental Fig. 4** TP53/FGFR3–NAC interaction plot for intratumoral CD4^+^ T-cell density

Interaction plot of intratumoral CD4^+^ T-cell density according to *TP53-* and *FGFR3-* mutation status and neoadjuvant chemotherapy (NAC) exposure. The interaction between *TP53* mutation status and NAC exposure was evaluated using a two-factor interaction model (interaction *p* = 0.0002).

**Supplemental Fig. 5** Representative image of HMGB1 expression

HMGB1 expression is positive in (a) NAC tissue but negative in (b) non-NAC tissue. NAC, neoadjuvant chemotherapy; HMGB1, high Mobility Group Box 1
